# Higher N stage and serum ferritin, but lower serum albumin levels are associated with distant metastasis and poor survival in patients with nasopharyngeal carcinoma following intensity-modulated radiotherapy

**DOI:** 10.18632/oncotarget.17418

**Published:** 2017-04-25

**Authors:** Xiaoqian Chen, Xianfeng Long, Zhongguo Liang, Hao Lei, Ling Li, Song Qu, Xiaodong Zhu

**Affiliations:** ^1^ Department of Radiation Oncology, Cancer Hospital of Guangxi Medical University, Nanning 530021, China; ^2^ Department of Radiation Oncology, Hubei Cancer Hospital, Wuhan 430079, China

**Keywords:** nasopharyngeal carcinoma, serum albumin, serum ferritin, distant metastasis, intensity-modulated radiotherapy

## Abstract

**Purpose:**

To evaluate the potential risk factors for distant metastasis of nasopharyngeal carcinoma in Chinese patients following standard intensity-modulated radiotherapy and chemotherapy.

**Methods:**

The potential risk factors for distant metastasis in 622 patients with newly-diagnosed primary nasopharyngeal carcinoma following standard radiotherapy and chemotherapy were evaluated retrospectively by stratification, univariate and multivariate analyses. The 5-year overall survival, distant metastasis-free survival, local recurrence-free survival and progression-free survival rates were determined.

**Results:**

Univariate and multivariate analyses indicated that N2-3 stage, serum ferritin > 300 μg/L and serum albumin < 42 g/L were independent risk factors for distant metastasis of nasopharyngeal carcinoma (*P* < 0.001, *P* = 0.013, *P* = 0.002, respectively). A risk prediction model was developed as follows: 1) low-risk group: 0-1 risk factor; and 2) high-risk group: 2-3 risk factors. Compared with low-risk group, the high-risk group had significantly lower 5-year distant metastasis-free survival (76.4% vs. 89.6%, *P* < 0.001), overall survival (76% vs. 85.9%,*P* < 0.001), local recurrence-free survival (88% vs. 92.4%, *P* = 0.029) and progression-free survival rates (68.2% vs. 83.7%, *P* < 0.001). In the high-risk group, patients with three risk factors had the lowest distant metastasis-free survival rate (*P* = 0.036).

**Conclusions:**

Combination of higher N stage, serum ferritin and lower serum albumin levels may be valuable for predicting distant metastasis of nasopharyngeal carcinoma patients following standard intensity-modulated radiotherapy and chemotherapy.

## INTRODUCTION

Nasopharyngeal carcinoma (NPC) is a malignant tumor with distinct regional distribution. NPC is a relatively rare disease in Europe and the United States with an incidence rate of < 1%. However, it is endemic in Southeast Asia, especially in South China [1, 2]. NPC is characterized by a poorly differentiated tumor at a complex anatomical location and easy invasion of adjacent organs. NPC is sensitive to radiotherapy, and NPC patients are usually treated by radiotherapies. While the therapeutic efficacy of radiotherapies for NPC patients, particularly with intensity-modulated radiotherapy (IMRT), has been recently improved following the advance in radiotherapy technology and equipment, the distant metastasis (DM) remains the major challenge for long-term survival [3–6]. Actually, even after combination of IMRT with neoadjuvant chemotherapy, concurrent or adjuvant chemotherapy, the DM rate still reaches about 20% [7–9]. Therefore, identification of DM relevant risk factors will be of great significance in reduction and prevention of DM in NPC patients and improving their overall survival (OS).

Currently, the TNM staging system has been demonstrated to have prognostic value and is used for guiding treatment strategies. However, even within the same TNM stage, varying OS periods and different risks for DM among NPC patients have been observed [10, 11]. Thus, simple TNM stage may be insufficient and ineffective to predict DM and discovery of other risk factors is necessary to build a more efficient prediction model to evaluate the DM risks in NPC patients.

Recent studies have showed that factors affecting the outcomes of cancer patients include the tumor characteristics and the host response factors [12–14]. The levels of serum albumin (ALB) are closely related to the degrees of malnutrition [15], affected by inflammatory responses [16], and associated with a poorer prognosis in cancer patients [17, 18]. In recent years, the levels of serum ALB are often combined with other inflammatory response indexes to evaluate prognosis in NPC [19, 20].

Serum ferritin (SF) is the primary intracellular iron-storage protein, regulating many physiological and pathological processes. Elevated SF levels have been recognized as a predictor of treatment response and are associated with poor prognosis in various malignancies, such as pancreatic cancer, non-small cell lung cancer, hepatocellular carcinoma and breast cancer [21–24]. However, there is rare study on the association between the levels of SF and DM in NPC patients [25].

The present study aimed to detect the potential risk factors for DM in Chinese patients with NPC and evaluated the prognostic value of abnormal levels of serum ALB, SF and other selective markers for DM besides the TNM stage in NPC patients following IMRT and chemotherapy.

## RESULTS

### Patient characteristics and treatment outcomes

To determine the potential risk factors for DM, 622 patients with NPC were recruited and treated with standard IMTR and chemotherapy. Their demographic and clinical characteristics are shown in Table 1. Those patients were characterized by higher frequency of male, patients with NPC at advanced stages of Union for International Cancer Control/ American Joint Committee on Cancer (UICC/AJCC), normal blood hemoglobin (Hb), but lower serum lactate dehydrogenase (LDH) and abnormal lower blood platelet (PLT) counts (Table 1). Patients were followed up with a median follow-up period of 43 months (range from 1 to 95 months), except for 12 patients, who were lost to follow-up. During the follow-up period, 18.3% of patients (114/622) had died (106 cases died of tumor, 2 cases died of severe nasopharyngeal bleeding and 6 cases died of other diseases), and 23% of patients (143/622) experienced tumor progression, including 9.5% (59/622) with local or regional recurrence and 16.1% (100/622) with DM. The 5-year OS, distant metastasis-free survival (DMFS), local recurrent-free survival (LRFS) and progression-free survival (PFS) rates were 82.9%, 83.9%, 90.8% and 77.3%, respectively.

**Table 1:** The demographic and clinical characteristics of patients

Clinical characteristics	Case numbers (%)	DM (%)	*P* values
All cases	622	100	
Age (years)			0.232
≥44	333 (53.53)	59 (17.71)	
<44	289 (46.47)	41 (14.18)	
Gender			0.521
Male	463 (74.44)	77 (16.63)	
Female	159 (25.56)	23 (14.47)	
2010UICC/AJCC T category			0.012
T1-3	433 (69.61)	59 (13.63)	
T4	189 (30.39)	41 (21.69)	
2010UICC/AJCC N category			<0.001
N0-1	287 (46.14)	27 (9.41)	
N2-3	335 (53.86)	73 (21.79)	
2010UICC/AJCC stage			<0.001
I	13 (2.10)	0 (0)	
II	120 (19.29)	8 (6.67)	
III	277 (44.53)	45 (16.25)	
IVA-B	212 (34.08)	47 (22.17)	
Hb (g/L)			0.208
≥120	546 (87.78)	84 (15.38)	
<120	76 (12.22)	16 (21.05)	
PLT(k/cc)			0.418
>300	107 (17.20)	20 (18.69)	
≤300	515 (82.80)	80 (15.53)	
ALB (g/L)			0.006
≥42	345 (55.47)	43 (12.46)	
<42	277 (44.53)	57 (20.58)	
LDH (U/L)			0.848
>245	59 (9.49)	10 (16.95)	
≤245	563 (90.51)	90 (15.99)	
SF (μg/L)			0.018
>300	251 (40.35)	51 (20.32)	
≤300	371 (59.65)	49 (13.21)	

Hb: hemoglobin, PLT: platelet, ALB: albumin, LDH: lactate dehydrogenase, SF: serum ferritin

### Prognostic factors for distant metastasis

Stratification analyses indicated that the percentages of DM in the patients with NPC at UICC/AJCC T4, N2-3, higher UICC/AJCC stages, lower serum ALB or higher SF levels were significantly higher than those with lower UICC/AJCC T, lower N or UICC/AJCC stages, higher serum ALB or lower SF in this population (*P* = 0.012, *P* < 0.001, *P* < 0.001, *P* = 0.006 and *P* = 0.018, respectively, Table 1). Univariate analysis revealed that NPC at T4 stage (*P* = 0.006), N2-3 stage (*P* < 0.001), serum ALB < 42 g/L (*P* = 0.004) and SF > 300 μg/L (*P* = 0.022) were significantly risk factors associated with DM (Table 2). Multivariate analysis further validated that N2-3 stage, serum ALB < 42 g/L and SF > 300 μg/L were independent risk factors for DM (*P* < 0.001, *P* = 0.002, *P* = 0.013, respectively).

**Table 2:** The risk factors are associated with distant metastasis in NPC patients

Variables	Univariate analysis			Multivariate analysis		
	HR	95% CI	*P* value	HR	95% CI	*P* value
Age, years (≥44 vs <44)	1.322	0.888∼1.970	0.170			
Gender (Male vs Female)	1.190	0.747∼1.895	0.465			
T staging (T4 vs T1-3)	1.756	1.179∼2.616	0.006			
N staging (N2-3 vs N0-1)	2.507	1.612∼3.899	<0.001	2.423	1.555∼3.775	<0.001
Hb, g/L (<120 vs ≥120)	1.407	0.824∼2.401	0.211			
PLT, k/cc (>300 vs ≤300)	1.262	0.773∼2.060	0.352			
ALB, g/L (<42 vs ≥42)	1.798	1.210∼2.671	0.004	1.774	1.182∼2.664	0.002
LDH, U/L (>245 vs ≤245)	1.125	0.585∼2.162	0.725			
SF, μg/L (>300 vs ≤300)	1.583	1.069∼2.342	0.022	1.647	1.111∼2.442	0.013

Abbreviations: Hb: hemoglobin, PLT: platelet, ALB: albumin, LDH: lactate dehydrogenase, SF: serum ferritin

### Risk prediction model for distant metastasis and survival

**Figure 1:**
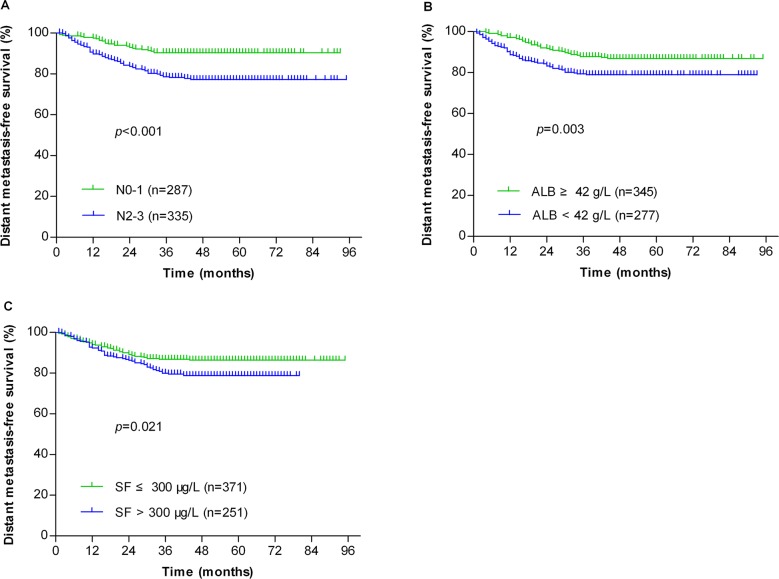
Kaplan-Meier analysis of the distant metastasis-free survival for all NPC patients (n=622) after being stratified by N stage **(A)**, serum albumin **(B)** and serum ferritin **(C)**.

**Figure 2:**
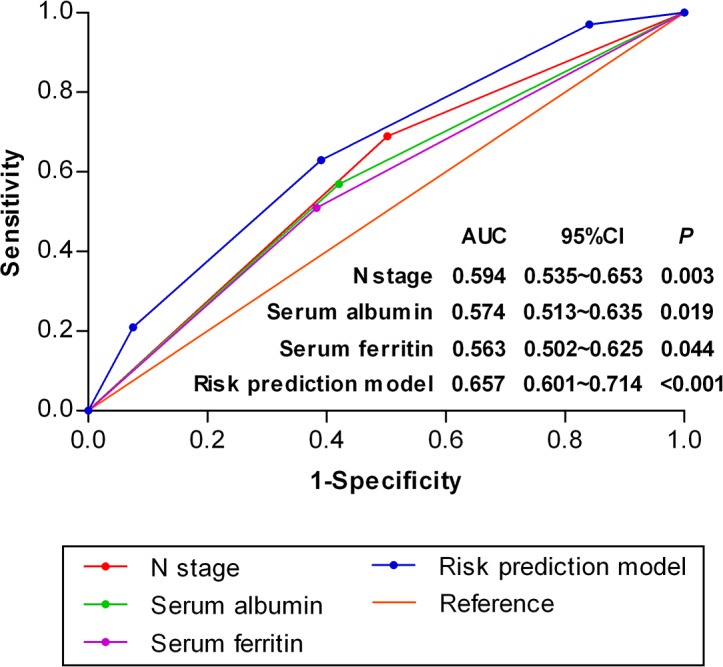
Receiver operating characteristic curves for distant metastasis in NPC patients (n=622) based on the individual risk factors and risk prediction model

**Figure 3:**
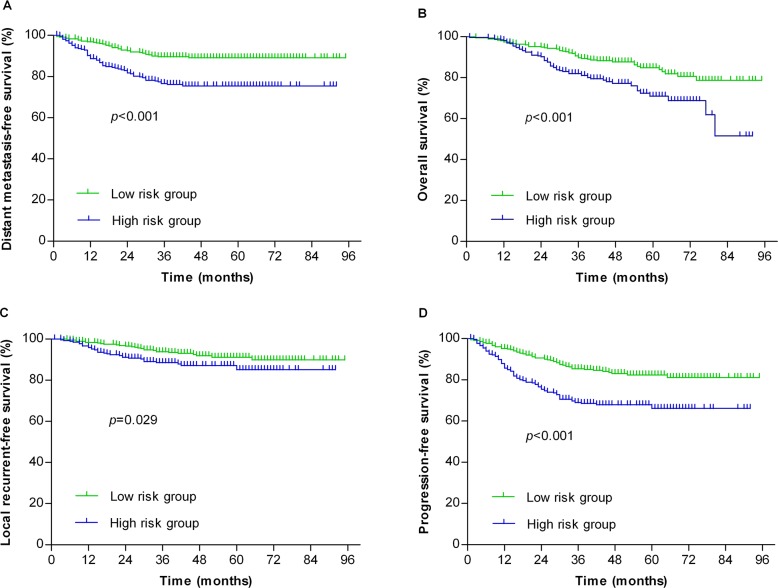
Stratification analysis of the survival of NPC patients The patients were stratified into the low-risk (n=355) and high-risk (n=267) groups using the distant metastasis risk prediction model. The distant metastasis-free survival **(A)**, overall survival **(B)**, local recurrent-free survival **(C)** and progression-free survival **(D)** were statistically analyzed.

Patients were stratified by individual risk factors of N stage, serum ALB and SF, and the DMFS rates of patients with NPC at N2-3, serum ALB <42 g/L or SF > 300 μg/L were significantly lower than the corresponding patients without the specific risk (Figure 1, *P* < 0.001, *P* = 0.003 and *P* = 0.021, respectively). Based on the three risk factors, a risk prediction model for DM in NPC patients was established. Patients were stratified into 1) low-risk group: 0-1 risk factor (355 patients); and 2) high-risk group: 2-3 risk factors (267 patients). The receiver operating characteristic (ROC) curves were used to evaluate the prognostic value of each risk factor and the risk prediction model (Figure 2). The 5-year DMFS, OS, LRFS and PFS rates of patients with high-risk were significantly lower than those with low-risk (76.4% vs. 89.6%, *P* < 0.001 for DMFS; 76% vs. 85.9%, *P* < 0.001 for OS; 88% vs. 92.4%, *P* = 0.029 for LRFS; 68.2% vs. 83.7%, *P* < 0.001 for PFS, Figure 3).

**Figure 4:**
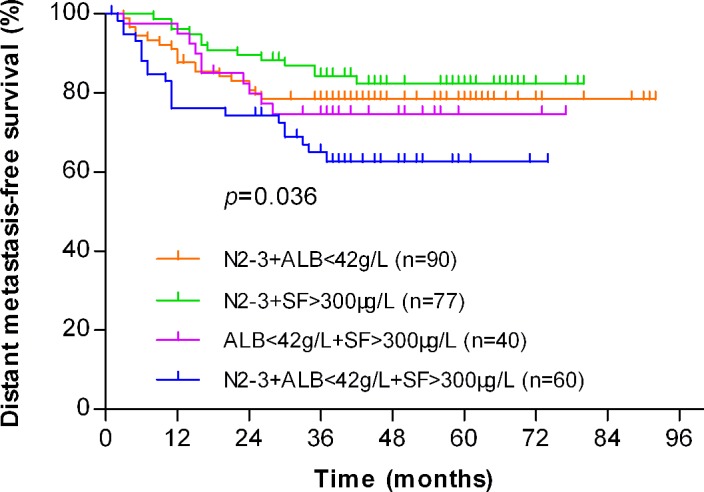
Kaplan-Meier analysis of the distant metastasis-free survival (DMFS) for NPC patients in the high-risk (n=267) group stratified by combinations of different risk factors The DMFS rate of patients at N2-3 stage and SF > 300 μg/L (n=77) had the best value, followed by patients at N2-3 stage and ALB < 42 g/L (n=90), and then the patients with ALB < 42 g/L and SF > 300 μg/L (n=40), while the patients with three risk factors (n=60) had the worst survival value (*P*=0.036).

In the high-risk group, there were 90 patients at N2-3 stage and ALB < 42 g/L, 77 patients at N2-3 stage and SF > 300 μg/L, 40 patients with ALB < 42 g/L and SF > 300 μg/L, and 60 patients with the three risk factors. There was a significant difference in the DMFS rates among these four groups (*P* = 0.036, Figure 4). Among them, the DMFS rate of patients at N2-3 stage and SF > 300 μg/L had the best survival value, followed by patients at N2-3 stage and ALB < 42 g/L, and then the patients with ALB < 42 g/L and SF > 300 μg/L, while the patients with three risk factors had the worst survival value.

## DISCUSSION

Local recurrence and DM are the main causes of mortality in M0 stage NPC patients following standard IMRT and chemotherapy. The IMRT is the first line of therapy for NPC and has significantly improved recurrence-free survival [26, 27], although DM remains the primary factor for mortality. In this study, we evaluated the potential risks for DM of NPC. We found that patients at N2-3 stage, SF > 300 μg/L and serum ALB < 42 g/L were independent risk factors of DM. Generally, T stage is closely related to local relapse, while higher N stage is connected with DM of NPC [28, 29]. Higher N stage was a risk factor for DM of NPC, consistent with previous observations [7, 30–32]. It is possible that NPC at an advanced N stage may have a higher risk for micrometastasis, which may be difficult to be overcome by an increase of irradiated dose to tumor targets by IMRT, leading to recurrence and DM [7].

In the present study, we found that NPC patients with lower serum ALB levels and higher SF were associated with an increased risk for DM, leading to a significantly lower DMFS rates in this population. Serum ALB is usually used to evaluate the long-term nutritional status and is affected by systemic inflammatory responses because ALB can promote wound healing and hormone synthesis [33]. Our findings were consistent with previous observations that lower serum ALB increases the risk of tumor progression and poor survival in patients with various cancers [34–36]. In NPC patients, the major pathologic type is undifferentiated non-keratinizing carcinoma, formerly known as “lymphoepithelial carcinoma”. This pathologic type of NPC contains numerous lymphocytes within the tumor, which indicates that inflammation may promote the development and progression of NPC [20]. Furthermore, the lower ALB and malnutrition may weaken the ability of patients to confront stress and anticancer therapy, hence deteriorating the life quality and survival [37]. Indeed, we found that NPC patients with lower serum ALB had significantly lower DMFS rate. Hence, correction of lower serum ALB may support the survival of patients with NPC.

SF is the best single marker to reflect the iron storage in vivo [38, 39]. As an ion transporter, iron participates in lipid peroxidation, leading to DNA damage [40, 41]. Previous studies have shown that excessive iron can suppress the function of CD4+ T cells [42], as well as the tumorcidal activity of macrophagocytes and monocytes [43, 44], but increase the numbers and activities of suppressor CD8+ T cells, impairing anti-tumor responses [45, 46]. The imbalance of immune function, together with higher plasma concentration of Epstein-Barr virus DNA, may be associated with an increased risk for DM in NPC patients [47–49]. Ferritin is a cytoplasm protein and responsible for regulating iron store and load. Currently, there is no clear information on what causes high levels of SF in a condition without iron overload [50]. First, the damage of red blood cells can release ferritin, leading to an increase in the levels of SF. Second, the tumor-associated oxidative stress and inflammation, such as chronic infection, can damage different types of cells to release ferritin to increase SF levels in a hemolysis-independent manner [51]. In addition, hepatic dysfunction and mucositis may also increase the release of ferritin to increase SF levels [52]. We are interested in further investigating what factors are associated with higher levels of SF in NPC patients.

The TNM staging system has been used for prognosis and treatment guidance in NPC patients, but the efficacy of its prognosis of DM is low. For example, patients with NPC at stage T1N2M0 may have a higher risk of DM than those at T3N0M0 stage, and they may require more aggressive treatments to reduce DM. In addition, the physical condition in individual patients also affects DM following initial treatments. Hence, combination of multiple factors to predict the risks of DM may valuable in management of NPC patients. Indeed, we combined independent risk factors of higher N stage, higher levels of SF and lower levels of serum ALB for DM to establish a predictive model. Stratification analysis indicated that NPC patients at a low-risk had significantly higher OS, DMFS, LRFS and PFS rates than those at a high-risk while patients with three risk factors had the poorest DMFS. Therefore, this prognostic model may be superior to the TNM classification system for evaluating DM of NPC.

We recognized that this study had limitations, such as a relatively smaller sample size, a single-institution retrospective analysis, which may have a selection bias to a certain extent. A prospective study will be necessary to confirm the reliability of this prognostic model.

In conclusion, our data indicated that a higher N stage, SF and lower levels of serum ALB were independent risk factors associated with lower OS, DMFS, LRFS and PFS rates in NPC patients. The risk prediction model based on these three risk factors may be valuable for physicians to evaluate potential risks of DM and to use for personalized medicine in NPC patients.

## MATERIALS AND METHODS

### Patients

A total of 622 NPC patients that had been treated with standard IMRT and chemotherapy at the Cancer Hospital of Guangxi Medical University were recruited from 2157 NPC patients between January 2007 and July 2012 and were analyzed retrospectively. The inclusion criteria included individuals with histologically confirmed NPC in nasopharyngeal biopsied sample; no evidence of DM; no previous history of a malignancy or other concomitant malignant disease; no previous treatment for NPC; Karnofsky performance status of 70 or more. Individual NPC patients, who did not meet the criteria, were excluded. Written informed consent was obtained from individual patients and the experimental protocol was approved by the Ethics Committee of the Cancer Hospital of Guangxi Medical University (Nanning, China).

### Laboratory measurements

The levels of blood Hb, PLT, serum ALB, LDH and ferritin in individual patients were measured before radiotherapy. The levels of blood Hb and PLT were measured using an automatic hematology analyzer (LH750, Beckman Counlter, USA). The levels of serum ALB, LDH and ferritin were measured using an automatic biochemical analyzer (7600-020, Hitachi High-Technologies, Japan).

### Radiotherapy

According to International Commission on Radiation Units and Measurements Reports 50 and 62, the target volumes and organs at risk in individual patients were delineated. All patients received one fraction of IMRT daily for 5 consecutive days per week. The prescribed radiation doses for all patients were 69.96-74.09 Gy at 2.12-2.39 Gy/fraction for 29-33 fractions to the planning target volume (PTV) of GTVnx (primary nasopharyngeal gross tumor volume) and GTVnd (involving cervical lymph nodes), which were 60-65.1 Gy to the PTV of CTV1 (high-risk regions), and 51.62-57.6Gy to the PTV of CTV2 (low-risk regions and neck nodal regions).

### Chemotherapy

In addition, 90.2% (561/622) of patients received neoadjuvant chemotherapy, concurrent or adjuvant chemotherapy. The neoadjuvant chemotherapy consisted of two or three cycles of both docetaxel (60 μg/m^2^), cisplatin (60 μg/m^2^ ) on day 1 and 5-fluorouracil (600 μg/m^2^/day) by continuously intravenous infusion for 5 consecutive days or docetaxel (75 μg/m^2^) and cisplatin (80 μg/m^2^) every three weeks. During the course of radiotherapy, the patients were treated with cisplatin (100 μg/m^2^) every three weeks or 40 μg/m^2^/week as concurrent chemotherapy. Adjuvant chemotherapy included two or three cycles of cisplatin (80 μg/m^2^) on day 1 and 5-fluorouracil (750 μg/m^2^) daily for 4 consecutive days every four weeks.

### Follow-up

The patients were followed up every 3 months during the first 2 years, every 6 months for the next 3 years, and then annually thereafter until death. The OS, DMFS, LRFS and PFS of patients were recorded.

### Statistical analysis

The statistical analyses were performed using the statistical package SPSS 16.0 (SPSS, Chicago, IL, USA). Patients were stratified, according to the levels of blood Hb, PLT and serum LDH, as previous descriptions [53–55]. Serum ALB and SF were analyzed as a binary variable using ROC curves to determine their cut-off values. When the area under the curve was the largest, the corresponding number was the cut-off value. The difference in the frequency of each group in individual category was analyzed by the Chi-square test. The survival of each group of patients was estimated by the Kaplan-Meier method and analyzed by the log-rank test. Individual risk factors for the DM of NPC in those patients were determined by univariate and multivariate analyses using the Cox regression model, including the hazard ratios (HR) and 95% confidence interval (95% CI). A two-sided *P*-value of < 0.05 was considered statistically significant.

## Abbreviations

DM: distant metastasis
NPC: nasopharyngeal carcinoma
IMRT: intensity-modulated radiotherapy
OS: overall survival
DMFS: distant metastasis-free survival
LRFS: local recurrence-free survival
PFS: progression-free survival
UICC/AJCC: Union for International Cancer Control/ American Joint Committee on Cancer
ALB: albumin
SF: serum ferritin
Hb: hemoglobin
LDH: lactate dehydrogenase
PLT: platelet
PTV: planning target volume
ROC: receiver operating characteristic
HR: hazard ratios.
